# Technical aspects of virtual augmented reality-based rehabilitation systems for musculoskeletal disorders of the lower limbs: a systematic review

**DOI:** 10.1186/s12891-022-06062-6

**Published:** 2023-01-03

**Authors:** Shamim Kiani, Iman Rezaei, Sanaz Abasi, Somayyeh Zakerabasali, Azita Yazdani

**Affiliations:** 1 Student Research Committee, Shiraz University of Medical Sciences, Shiraz, Iran; 2 Physical Therapy Department, School of Rehabilitation Sciences, Shiraz University of Medical Sciences, Shiraz, Iran; 3 Rehabilitation Sciences Research Center, Shiraz University of Medical Sciences, Shiraz, Iran; 4 Health Human Resources Research Center, Shiraz University of Medical Sciences, Shiraz, Iran; 5 Health Information Management Department, Shiraz University of Medical Sciences, Shiraz, Iran; 6 Clinical Education Research Center, Shiraz University of Medical Sciences, Shiraz, Iran

**Keywords:** Virtual reality, Augmented reality, Rehabilitation, Musculoskeletal disorder, Lower limb

## Abstract

**Introduction:**

Musculoskeletal disorders are one of the most common causes of physical disability. The rehabilitation process after musculoskeletal disorders is long and tedious, and patients are not motivated to follow rehabilitation protocols. Therefore, new systems must be used to increase patient motivation. Virtual reality (VR) and augmented reality (AR) technologies can be used in this regard. In developing such systems, various technologies and methods of movement recognition are used; therefore, this study aims to summarize the technical aspects of using VR/AR in rehabilitation and evaluate and discuss efficient methods of investigating studies using the Statement of Standards for Reporting Implementation Studies (StaRI).

**Methods:**

Search in four scientific databases was done systematically based on PRISMA through online search engines from inception to June 2021. These databases include Medline (PubMed), Scopus, IEEE, and Web of Science. An updated search was also conducted on 17 December 2021. The research used keywords and MeSH terms associated with VR/AR, musculoskeletal disorder, and rehabilitation. Selected articles were evaluated qualitatively using the Standards for Reporting Implementation Studies (StaRI) statement.

**Results:**

A total of 2343 articles were found, and 20 studies were included. We found that 11 (55%) studies used Kinect technology as input tools, and 15 (75%) studies have described the techniques used to analyze human movements, such as dynamic time warping (DTW) and support vector machines (SVM). In 10 (50%) studies, the Unity game engine was used for visualization. In 8 studies (40%), usability was assessed, and high usability was reported. Similarly, the results of the review of studies according to the StaRI checklist showed poor reporting in the title and discussion of the studies.

**Conclusions:**

We found that academic studies did not describe the technical aspects of rehabilitation systems. Therefore, a good description of the technical aspects of the system in such studies should be considered to provide repeatability and generalizability of these systems for investigations by other researchers.

## Introduction

Musculoskeletal disorders are one of the most common causes of physical disability and long-term pain [1]. World Health Organization (WHO) states that; musculoskeletal disorders affect the muscles, bones, joints, and related tissues such as tendons and ligaments [2]. The incidence of musculoskeletal disorders is increasing, with a prevalence rate of 25% [3]. Musculoskeletal disorders of the lower limbs are common in a variety of contexts, such as sports injuries (such as anterior cruciate ligament) [4], knee and hip injuries (such as osteoarthritis and joint replacement) [5], and ankle instability [6, 7]. Joint and muscle problems in the lower limbs reduce walking efficiency [8]. Lower limb rehabilitation helps to restore the patient’s natural movement and function, such as standing and walking [9]. There are many guidelines for better management of musculoskeletal disorders [10]. One of these methods is exercise therapy [3, 6], which can be defined as a physical activity program involving muscle contraction and body movement to relieve symptoms and improve function [11].

Exercise therapy can improve symptoms and daily functioning in people with these disorders, but treatment results often prove ineffective [3]. Considering the treatment takes and is repetitive, patients may choose not to adhere to it, which can lead to treatment failure [12]. Also, continuous access to rehabilitation centers is limited for various reasons, including cost, geographical location, and wasting time commuting and waiting in rehabilitation centers [6]. According to the Global Burden of Disease estimates from 1990–2019, as the world population ages and economic and social inequalities increase, musculoskeletal disorders may increase in the future [13, 14]. Digital technologies can cover part of healthcare remotely using virtual care and maximizing the efficiency of health care delivery [15, 16].

Two digital technologies can be used for virtual care services: virtual reality (VR) and augmented reality (AR). VR/AR can be a complementary treatment tool for rehabilitation and physiotherapy [17]. Using these technologies makes it easier to do repetitive exercises and provides a mechanism for encouraging patients through feedback [18]. VR/AR can assist in reducing the workload for specialists [17, 19]. VR makes remote rehabilitation possible [20]. Also, in an epidemic state, including COVID-19, VR can effectively solve or tackle many of the current challenges [21]. According to the study by Levac et al. [22], the primary purpose of using VR-based rehabilitation is to reduce musculoskeletal complications after a stroke (25.8%), brain injury rehabilitation (15.3%), musculoskeletal disorder (14.9%), cerebral palsy (10.5%), and neurological disorders (6.3%).

Various systematic studies have been conducted on the use of VR/AR in the rehabilitation of musculoskeletal disorders [19, 23–25]. Gumaa and Rehan Youssef [19] examined the effect of VR on orthopedic rehabilitation. Evidence from their study suggests the effectiveness of VR in chronic neck pain and shoulder impingement syndrome was promising. Gumaa et al. [23] also evaluated the validity and reliability of VR games and real-time feedback in assessing the musculoskeletal system. Based on the results, there is limited promising evidence that interactive VR using games or real-time feedback is very reliable in the range of motion (ROM) assessment in asymptomatic participants and patients with chronic neck pain and radial fracture. However, the evidence for solid conclusions for other diseases is limited.

In their study, Ayed et al. [26] focused on using serious games and vision-based VR systems for motor rehabilitation. Serious games focus on problem-solving instead of entertainment and help people understand different topics [24]. Their purpose was to provide a research method engineers can use to improve their clinical trials’ design and reporting processes. The findings show that patients with cerebral palsy and stroke are the main target groups in this area, and special attention has been paid to elderly patients [26]. Byra and Czernicki [25] evaluated the effect of VR in rehabilitating elderly patients with osteoarthritis of the knee or hip, including patients after arthroplasty. Their study included ten randomized controlled trials focusing on the use of games and biofeedback in the rehabilitation of patients with osteoarthritis of the knee and hip. They stated that the effectiveness of VR-based rehabilitation was uncertain and that evidence for patients after complete hip arthroplasty was scarce.

Vinolo Gil et al. [17] studied the scientific evidence for AR treatment complementing physiotherapy and the most effective methods. They stated that AR, in combination with conventional therapies, had a positive effect on physical function in the elderly, lower and upper limb function in stroke, and phantom pain. VR/AR in rehabilitation has been increasingly researched, and the technology used in healthcare has changed dramatically. However, little information has been published about the technical features and how these features have changed over time. Addressing the required technical aspects can increase the probability of project success in the early implementation process [27]. Presenting the results of reviewing articles that provide VR/AR rehabilitation systems in technical detail provides repeatability and generalizability of these systems for studies by other researchers [27]. Developers can also work with the current literature and collaborate with physicians to design VR/AR systems that focus on rehabilitation.

The need to study the technical aspects in the field of e-health has been studied in some studies. For example, Fatehi et al. [28], in examining the technical aspects of clinical video conferencing, stated that the successful implementation of telemedicine requires the proper use of basic technology. Therefore, this study aims to summarize the technical aspects of using VR/AR in rehabilitation and evaluate and discuss efficient methods of investigating studies using the Statement of Standards for Reporting Implementation Studies (StaRI). To achieve these aims, we examine the following issues in VR/AR-based rehabilitation systems for lower extremity disorders. We are investigating VR/AR tools and sensors used in these systems. We are reviewing approaches for motion detection, examining VR/AR-based systems development tools, and evaluating the applicability of VR/AR-based systems to patients with lower limb musculoskeletal diseases. Lastly, we conduct a qualitative evaluation of studies that have developed lower limb rehabilitation systems.

## Methods

This literature review was based on the Preferred Reporting Items for Systematic Reviews and Meta-Analyses (PRISMA) guidelines [29].

This study is part of a research project entitled "Prototype design of the lower limbs rehabilitation game based on virtual reality" with the ethics code of IR.SUMS.REHAB.REC.1399.050, registered in https://research.ac.ir/, is a system of research projects in Iran.

### Search strategy

Systematic search through online search engines was conducted from inception to June 2021, included Medline (PubMed), Scopus, the Institute of Electrical and Electronics Engineers (IEEE), and Web of Science. An updated search was conducted on 17 December 2021. Comprehensive research was done using keywords and medical subject headings (MeSH) terms associated with VR/AR, musculoskeletal disorder, and rehabilitation. The search strategy and keywords are shown in Table 1. In addition, the reference lists and citations of the included articles were manually checked to identify other studies.

**Table 1:** Keywords and search strategy

Keywords	virtual reality, augmented reality, rehabilitation, musculoskeletal diseases, lower limb, physiotherapy
Search Strategy	("virtual reality" OR VR OR " video games" OR "serious game" OR "virtual environment" OR "interactive gam*" OR exergame OR "augmented reality" OR x-box OR Kinect OR Nintendo OR Wii) AND (( knee OR hip OR ankle OR leg) OR ("musculoskeletal diseases" AND "lower limb")) AND (physiotherapy OR exercise OR therapeutic OR treatment OR "exercise therapy" OR rehabilitation) NOT (stroke OR cerebral palsy OR cancer OR tumor OR carcinoma OR neurologic∗ OR dentistry)

The inclusion of articles was under the following criteria: 1) articles are written in English, 2) articles that considered adult participants with musculoskeletal disorders of the lower limbs, and 3) articles that were on the implementation and development of rehabilitation systems.

Exclusion criteria also included the following: articles that 1) were only for improving balance and gait analysis, 2) used exoskeletons or accessories for rehabilitation, 3) were about body anatomy simulators, 4) were about simulators for teaching orthopedic surgery, 5) describe VR/AR systems for treating pain in amputated patients, and 6) from VR/AR that were used for neurological disorders. Theses, book chapters, letters to editors, reports, and reviews were also excluded.

### Data collection process

Two independent reviewers (SH, SA) screened the retrieved studies by title and abstract. Articles that met our inclusion criteria were selected for the full-text screen. Any disagreement was resolved by discussion with the senior authors (AY, ZA, and IR). We summarized critical articles and entered them into customized extraction forms based on these categories to diminish bias. Lastly, specific categories were considered to classify and analyze the relevant articles after we selected the final articles. Two authors (SA and SK) independently extracted the study characteristics from each article based on the classification. The information extracted by the researchers was re-examined to reach an agreement. The reviewers (AY, ZA, and IR) assessed and verified the extracted data. EndNote X9 software was used for resource management. All syntheses and analyses were performed using SPSS v25.

The results were divided into the descriptions of studies, technologies, movement recognition and assessment, VR/AR systems development tools, evaluation of VR/AR-base systems, and quality evaluation of studies to achieve the objectives.

### Quality of studies

The purpose of the StaRI is to develop guidelines for transparent and accurate reporting of implementation and identify the shortcomings in reporting the existing studies. The StaRI was developed using the e-Delphi technique and an international consensus session [30].

The StaRI checklist comprises 27 items. This checklist is the concept of dual strands describing (a) the strategies used to promote implementation and (b) the intervention being implemented [30]. Also, using precise and clear criteria reduced the likelihood of diagnosis bias.

## Result

### Search results

**Fig. 1:**
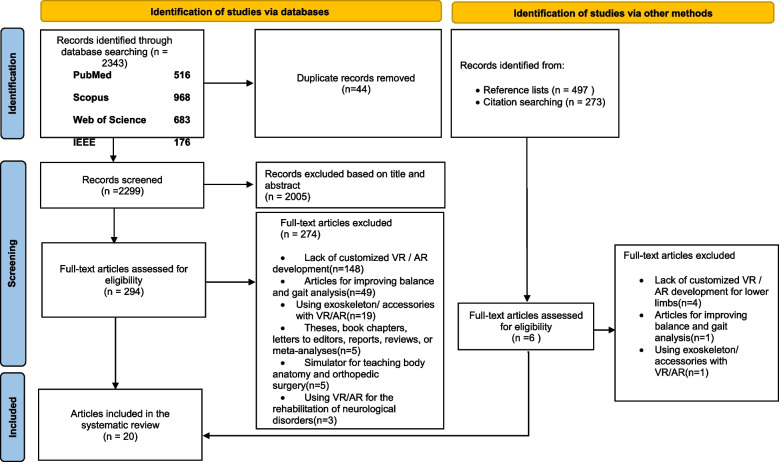
Literature search flow diagram

The initial search yielded 2343 studies from databases. After evaluating the title and abstract of the studies, based on our inclusion criteria, 294 articles were selected for evaluation of the full text. Finally, 19 journal articles met our inclusion criteria. A second search was conducted on 17 December 2021 to find new studies. Two recent studies were found for full-text analysis. Then, one new study was selected. The manual search did not add any new articles to the study. Figure 1 presents the PRISMA flowchart of the study selection.

### Description of studies

**Fig. 2:**
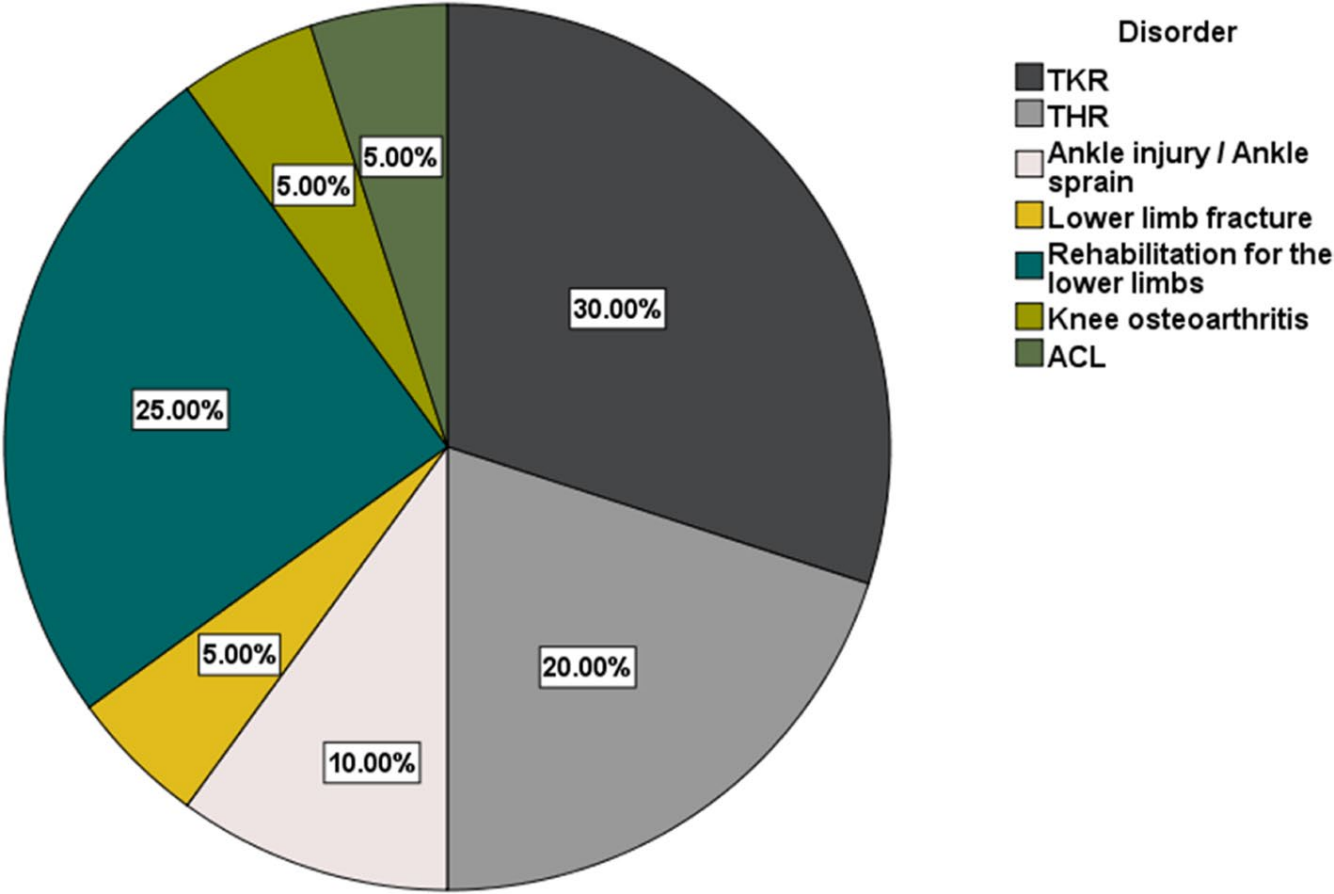
Frequency of the studies due to lower limb disorders

Of the selected studies, 10 (50%) were conference studies. According to Table 2, in 15 (75%) studies, in addition to implementation, the VR/AR evaluated the system, and the sample size was reported. The sample size ranged from 4 to 287. The age of the participants was reported in 9 (45%) studies, and the participants were over 18 years old. As shown in Fig. 2, 6 (30%) studies focused on total knee replacement (TKR) rehabilitation, 5 (25%) rehabilitation for the lower limbs, 4 (20%) total hip replacement (THR) rehabilitation, 1 (5%) after lower limb fracture surgery rehabilitation, 2 (10%) ankle injury rehabilitation, 1 (5%) anterior cruciate ligament (ACL) rehabilitation, and 1 (5%) knee osteoarthritis rehabilitation.

**Table 2:** The characteristics of the reviewed articles

#	**Author** **/ Year**	**Research objective**	**Result**	**Type disorder** **/Sample size**	**Rehabilitation exercises**	**VR/AR tools and sensors**	**Tools develop**	**Movement Recognition and Assessment**	**Save data**	**Feedback**	**Evaluation methods**
1	Guggenberger et al. [31]/2021	The development was a game for mobility and lower limb strength through bodyweight training in an AR environment	The results show that the game under review has the potential of a low-cost, affordable approach to supporting patients in their home exercise program after THR and completing inpatient rehabilitation	THR People:30	Squats Step-ups Side-steps	HMD Samsung Galaxy S9/ Huawei P20 smartphone Implements the smartphone’s built-in IMU 3DMAS	Google ARCore API	By calibrating the system, using camera images of the room, ARCore detects feature points, which are used as references for positioning virtual objects and in combination with the IMU data for motion tracking	NM	+	NM
2	Zhao et al. [32]/ 2021	Design and implement a system for rehabilitating people who have undergone total knee replacement surgeries	This avatar-based interactive system facilitates rehabilitation exercises at home. They showed that an integrated system could track movements and instantly assess the quality and quantity of movements using XML-defined rules for TKR rehabilitation	TKR	-Namely -Quad set -Side-lying hip abduction -Straight raise leg -Ankle pump	IMU/Kinect	-Unity 3D -SDK -C# program-ming language -XML	-Rule-based framework and the mechanism used to track the movements dynamically	NM	+	NM
3	Prvu Bettger et al. [33]/ 2019	They evaluated the clinical outcomes and cost of the virtual PT program compared to traditional care after TKR	Virtual PT with remote rehabilitation reduces the cost of 3 months of health care after TKR while having similar effectiveness compared to the traditional rehabilitation method	TKR People: 287 Mean age: 65 years	-Knee extension -Knee flexion -Gait speed -Safety measures	Kinect	-SDK -Microsoft visual studio 2010 in the Windows 7 operating system -C# programming language	NM	+	+	-KOOS -PROMIS -Satisfaction with physical function -Physical activity
4	Günaydin and Arslan [12]/ 2019	This paper presents a low-cost rehabilitation support system for lower limb muscle strengthening exercises	This study has successfully developed a system to increase patient participation in treatment and provide physiotherapist tools for remote follow-up	Rehabilitation for the lower limbs People:16	-Active knee extension exercises to strengthen the quadriceps muscle -Active knee flexion for hamstring muscle strengthening -Terminal extension exercise for vastus medialis obliquus muscle	sEMG (BITalino)	-Unity 3D -APIs provided on the BITalino website	Feature extraction methods are used for electromyography signal analysis	+	+	-Perceived Usefulness (PU) -Perceived Ease of use (PE) -Attitude (AT) -Intention of use (IU)
5	Perez Medina et al. [34]/ 2019	This paper presents a flexible, scalar, and modular software architecture for the ePHoRt platform	The main role of this work is to act as a conceptual guide for developing remote rehabilitation operating systems	THR	-Hip abduction -Slow flexion of hip and knee -Hip extension -Frontward, sideway, backward	Kinect	-Django 2.0.5 -Model-view-controller (MVC) -CSS -Boostrap 4.1.13 -JQuery 3.1.14 -RESTful API -Kinectron 1.4.2 -Processing 1.4.8 -JavaScript Library/ Three.jsr97	-The evaluation of the movement quality is carried out by an AI algorithm based on the DTW algorithm -A SVM algorithm was trained to detect the face and process it based on the following criteria	+	+	The patients must answer a preliminary questionna-ire regarding their health status. The preliminary questionnaire allows the physiotherapist to get relevant patient information, like recent surgeries, levels of pain, and other health problems
6	^a^Kontadakis et al. [35, 36]/ 2017–2018	This paper presents the design and implementation of a custom rehabilitation program for patients undergoing TKR and encourages the patient to exercise	Initial testing of the application indicates that patient engagement is enhanced in most cases	TKR 1-People: 6 Age: 30–50, 64–80 2-People: 10 Age: 64–80	The selected exercise for testing was knee extension	IMU	-Python programming language -The Java native interface -C# programming language -Unity 3D	This study proposes a new classification algorithm that automatically classifies an exercise in real-time as true or false	+	+	ROM
7	Karayigit and Celikcan [37]/ 2018	This paper presents the design and implementation of knee up, a game to increase knee health	Study results demonstrate that knee-up is generally well-received in terms of usability, engagement, ease of learning-to-play, and exercise sustainability; and validate that the rule-based recognition algorithm works satisfactorily well	Rehabilitation for the lower limbs People: 20 Mean age: 37.6	Standing Knee Raises Movement (SKRM)	Kinect	- Unity 3D -XML	Implementation of rule-based SSP and SKRM recognition algorithm	+	NM	-SSP takes the static skeletal measurements required for the recognition algorithm -Questionn-aire 11 questions on a Likert scale of 1 to 5 -ROM
8	Rybarczyk et al. [38]/ 2018	This paper presents the creation of a remote platform for self-motor rehabilitation and remote monitoring by health professionals to improve patients after hip replacement	The results show high accuracy in evaluating the movements (92% of correct classification). The overall SUS score is 81 out of 100, which suggests a good usability	THR People: 22 Age: 20–50	NM	Kinect	-Django technology -Python -Node.js	Hidden Markov models approach	+	+	-SUS -Before the rehabilitation begins, patients must answer a questionnaire, which evaluates their ability to complete the exercises
9	Feng et al. [39]/ 2018	In this paper, a VR-based training system for ankle joint rehabilitation is designed	The experimental results verify the feasibility of the system. The system designed in this paper offers significant potential benefits in rehabilitation but remains to be properly explored	Ankle injury People: 5	Six types of ankle joint movements	EMG MPU6050 HMC5883	-C + + language	This paper proposes a rehabilitation assessment method based on the multi-index fusion of kinematics and EMG signals. The method includes three indexes: root means square of EMG, joint activity, and joint smoothness	+	+	-RMS -Joint activity -Joint smoothness
10	Pruna et al. [40]/ 2017	The 3D virtual system is presented for the rehabilitation of lower limbs also, and two games are designed to allow flexion, extension, and strengthening movements	They show that the systems have a good acceptance for rehabilitation	Knee osteoarthritis People: 4 Age: 61–78	Allow the execution of flexion and extension movements as well as knee strengthening	-3 Space Mocap Sensors -YEI 3-Space Mocap movement capture devices - 6 sensors (3 for the left leg and 3 for the right leg)	-Unity 3D -Scripts were developed for the system design	NM	NM	+	SEQ usability test
11	^a^Tannous et al. [41, 42]/ 2015–2016	This paper presents the feasibility of using a serious new game system to improve lower limb musculoskeletal rehabilitation	According to the results, the squat was the most difficult challenge. In addition, any functional rehabilitation exercise’s performance depends on each participant’s physiological characteristics The game showed useful functions for various programs (home rehabilitation and exercise)	Rehabilitation for the lower limbs People: 10 Mean age:24.2 ± 3.79 years	-Easy category: hip adduction/abduction and straight jump exercises -Medium category: hamstring curls, the high knees and one-leg stance exercises, standing exercises -Hard category: Squat exercises	Kinect	-Microsoft XNA Game Studio (Washington, USA) -Visual Studio.Net with C#	Based on the position and direction of a particular joint, an angle can be calculated to stimulate the 3D avatar model. Two types of motion can be considered: single degree of freedom (DOF) motion and multiple DOF motion	+	+	-They designed a special questionnaire to get participants’ opinions on the developed system’s various aspects (games, exercises, and users)
12	Su and Cheng [43]/ 2016	This paper investigates the effect of achievement factors on patient rehabilitation after complete knee replacement (TKR) exercises using a game rehabilitation system based on the PCA-ANFIS model	The SUS shows that the PRS is acceptable The developed system allows patients to strengthen their self-efficacy through a game-based rehabilitation environment and allows them a faster and more complete recovery	TKR People: 34	kicking	Kinect	-Unity 3D	-A combination of PCA and ANFIS has been used to provide a predictive emotion model	NM	NM	- SUS - AKSS -SER scale -Demographic scale
13	Zhiyu et al. [44] /2015	This paper was to determine the feasibility of the Kinect in assisting subjects in performing a single posterior chain strengthening exercise	Game testing showed that our system is fast, accurate, robust, and works well for game control	ACL People: 6	-Jumping action by the side-lying hip abduction exercise	-Kinect -Marker-based vicon system	Unity3D	-Hough leg angle estimator (HE)	NM	NM	NM
14	Antón et al. [45]/ 2015	This paper presents the development of the Kinect rehabilitation system (KiReS)	Participants also found the interaction with Kinect easy and enjoyable, showing a predisposition to using the system again	THR People: 7 Mean age:56	Ten exercises for both the left and the right hip	Kinect	NM	They used an algorithm to detect movements, the two main elements of which are the position classification and the exercise recognition methods They use a variant of the DTW algorithm	+	+	Patients’ subjective perceptions: Likert scale questionnaire In three categories: the system, the user’s experience, and the interface
15	Choi et al. [46]/ 2015	A program was developed for leg-strengtheni-ng exercises and balance assessment using Microsoft Kinect	The developed program can enable users to conduct leg-strengtheni-ng exercises and balance assessments at home	Rehabilitation for the lower limbs People: 5 Mean age: 24.8 ± 2.9	-Three leg-strengthening exercises (knee flexion, hip flexion, and hip extension) – OLST	Kinect	-SDK -Microsoft visual studio 2010 in windows 7 -C#	-Calculating the angles at the hip and knee joints using the position data around the joints	NM	+	- OLST
16	Gonzalez-Franco et al. [47]/ 2014	This paper addressed the problem of patients’ adherence to physiotherapy and motivation using a VR game interface	Patients would be able to perform the whole therapy on their own at home. We can conclude that the system has been proven usable as all participants could start, follow and finish the treatment independently without external intervention	TKR People: 16 Mean age: 26.18	-The potentially familiar exercises included:(semi-recumbent heel slides, sitting knee extension-flexion, and a straight leg raise extracted from the rehabilitation protocols for Knee Arthroscopy Rehabilitation) -The unfamiliar exercises involved: (3 combinations of hip flexion and knee extension exercises)	-Wocket: three-axis positioning miniature wireless accelerometer	- Unity3D	NM	+	+	Ten points Likert -scale questionnaire to report: ( Interactions- Attention demanding- Learning- Control- Usability- Time perception)
17	Garcia and Felix Navarro [48]/ 2014	This paper focuses on developing the Mobile RehApp, an augmented reality-based mobile device application designed for therapeutic support that aims to assist physiotherapists and patients on ankle sprain rehabilitation	This implementation enhances user interaction as no game controllers or sensors are required to make the game intuitive and easy to use. More importantly, this implementation has the potential to be used as a means to provide home-based therapy, increasing levels of motivation and adherence to rehabilitation	Ankle sprain	ROM exercises	Custom-made AR marker	-Unity 3D -SDK	NM	+	+	NM
18	Patanapanich et al. [49]	This paper presents a physical rehabilitation system	The results show SPRS allows patients to rehab correctly with a slight error which is better than exercise without assistance from the software	Rehabilitation for the lower limbs	-Hip extension -Side leg raise -knee flexion -Hip flexion Plantar flexion -Tandem walk -Sidewalk Chair Stands -Timed Up&Go	Kinect	-OpenNI2 -NITE2 API - Visual C + +	Variables that used to detect movement	+	+	NM
19	^a^Shih-Ching et al. [50]; Yeh et al. [51]	This paper is intended to develop a lower limb fracture post-operative guided interactive rehabilitation training system for the hip, knee, and ankle joints and establish a method of motion analysis and a method of motion performance assessment in conjunction with wireless sensor technology and animation techniques	The medical evidence shows that a guided rehabilitation system can enhance the patient’s motivation and willingness to be committed to rehabilitation training and increase the quality and amount of exercise activity during the training process	After a fracture surgery	-Bending knee -Raise thigh	IMU(Gyro)	Unity 3D	The state values ​​of an object with IMU	+	+	NM
20	Maurer et al. [52]	This paper presents a prototype game as a controlled learning environment for home use	The prototype-controlled learning environment can provide more efficient and accurate rehabilitation	TKR	Knee-bend exercises	Kinect	NM	NM	+	+	NM

*NM* Not mentioned in the article

^a^These studies described a system in one version and evaluated it in another

According to Table 2, 17 (85%) systems developed for lower limb rehabilitation give feedback during or after exercise therapy. This feedback was either visual or a combination of visual and auditory feedback [12, 31–35, 38–41, 45–49, 51, 52]. Feedback in rehabilitation systems can help the patient better adapt to the treatment protocol [3].

### Technology

#### Kinect

Kinect, made by Microsoft for games, is a low-cost motion camera that can provide information about the 20 major human joints in three-dimensional (3D) coordinates. This information can develop various rehabilitation systems with Kinect [46, 53].

Among the studies, 11 (55%) used Kinect technology as input tools [33, 34, 37, 38, 41, 43–46, 49, 52].

#### Inertial measurement unit (IMU)

One approach to evaluating rehabilitation exercises is to use inertial sensors, which include IMU and magnetic sensors, accelerometers, and gyroscopes, which measure an object’s linear acceleration and angular velocity [54].

According to our findings, 2 (10%) studies used the IMU technology as an input tool [35, 36, 50].

Guggenberger et al. [31] used the built-in inertia measurement unit and the integrated front camera of the smartphone and head-mounted displays (HMD) to track the movements and produce the corresponding AR images. Furthermore, in 2021, Zhao et al. [32] used a combination of Kinect and IMU technologies for real-time rehabilitation and motion-tracking exercises. They boosted the system with the IMU sensor because the hip and knee angles can be significantly tracked with the Kinect, but tracking ankle movements is difficult with the Kinect.

#### Surface Electromyography (sEMG)

We found 2 (10%) studies used sEMG technology as input tools [12, 39]. In Günaydin’s study, a serious concept of computer games for physiotherapy and lower limb rehabilitation using sEMG signals and a feedback module for remote tracking of patients is presented. When the patient plays a game, the sEMG signals are recorded and then analyzed. Measuring sEMG during rehabilitation provides information about the progress of related muscles [12].

#### Other input tools

Three studies did not use the above input tools [40, 47, 48]. Pruna et al. [40] implemented a 3D virtual lower limb rehabilitation system using three space mocap sensors. Gonzalez-Franco et al. [47] used an accelerometer to empower patients in physiotherapy at home. Garcia and Felix Navarro [48] aimed at rehabilitating people with ankle sprains; they implemented an augmented reality application for mobile devices using an AR marker.

### Movement recognition and assessment

Providing a rehabilitation program through an interface that detects human movement can help to perform the correct movements [37].

Some studies have described the techniques used to analyze human movements. Here, recognition and assessment techniques were classified according to the sensors used.

#### Movement recognition with Kinect

Kinect can provide real-time, in-depth skeleton tracking information of 20 joints and red, green, and blue (RGB) images for movement recognition [46]. Among the studies, 2 (10%) used the dynamic time warping (DTW) algorithm to distinguish between right and wrong movements [34, 45]. This algorithm processes the skeletal data [34]. Another successful method for achieving movement recognition is the discriminative approach. The main classifiers that use this method to identify movements are k-nearest neighbor (KNN), support vector machines (SVM), naïve Bayes, and the C4.5 decision tree. The last two algorithms are the most popular because they allow high classification accuracy [55]. In addition to DTW, Perez Medina et al. [34] used an SVM algorithm to recognize and process faces.

In Tannous et al.’s study on the avatar scaling process, a linear rigid trans-formation was applied, and body height, computed from the Kinect, was used as a scaling factor [41]. Since the Kinect skeleton model did not provide a reasonable estimate of ground positions, Zhiyu et al. [44] used depth images Kinect and multiple leg angle estimators for different angle regions to recognize. Choi et al. [46] also used common Kinect data to detect leg-strengthening exercises. Rybarczyk et al. [38] used an evaluation module based on the Hidden-Markov model approach to assess the quality of real-time movements. Su et al. [43] used a combination of a principal component analysis (PCA) and an adaptive network-based fuzzy inference system (ANFIS). They provided a predictive emotion model-based artificial emotion model with a Plutchik emotional wheel.

One approach to recognizing human movements is the rule-based method. In this method, movements are first described based on a set of rules and then classified according to the rules set for each movement [56]. In two studies, the rule-based method was used to recognize the movements. These studies define all rules based on the extensible markup language (XML) [32, 37].

#### Movement recognition whit IMU

Kontadakis et al. [36] used an automated exercise classification algorithm using data from the IMU sensor to recognize the movements. The input data of the algorithm were filtered using a Complementary filter. The algorithm’s output was a computational decision for the correctness or otherwise of the exercises. In 2018, they also used a similar algorithm to recognize the movements [35].

#### EMG signal analysis

In 2 (10%) studies, when the patient was playing, EMG signals were stored and then analyzed for quantitative evaluation of rehabilitation, and feature extraction methods were used to analyze the EMG signal [12, 39]. Feng et al. [39] propose a rehabilitation assessment method based on the multi-characteristic fusion of kinematic signals and EMG. This method consists of three indicators: mean square root EMG, joint activity, and joint smoothness.

### VR/AR systems development tools

In 10 (50%) studies, including the AR study, the Unity game engine was used for visualization [12, 32, 35, 37, 40, 43, 44, 47, 48, 50]. Unity is a powerful and stable tool for designing and developing games, which has received much attention in the game industry [43]. We found that the non-commercial Kinect windows software development kit (SDK) and C# as the programming language for Kinect capabilities were the most common tools for developing rehabilitation systems [32, 33, 35, 41, 46, 48].

### Evaluation of VR/AR-base systems

Examining the studies, we found that six studies (30%) did not express the system evaluation method, and AR-based research was one of these studies [32, 44, 48–50, 52].

#### System effectiveness evaluation

Most studies did not perform clinical trials on developed systems and only examined the systems with an initial evaluation; this initial evaluation of the rehabilitation systems developed showed the promising impact of these systems on lower limb rehabilitation. In 4 (20%) studies, it was stated that VR-based rehabilitation had an important role in motivating patients, potentially leading to greater participation and better outcomes in rehabilitation [12, 35, 48, 50].

According to Table 2, some studies used rehabilitation assessment methods, including measuring the amount of ROM [35, 37], one-leg standing test (OLST)[46], knee injury and osteoarthritis outcome score (KOOS) [33], patient-reported outcomes measurement information system (PROMIS) [33], American knee society score (AKSS) [43], and a primary health status questionnaire [34].

#### System usability evaluation

Acceptance of the system by the user is critical. The two vital factors in adopting a system are examining the usability of the system and considering the principles of user-centered design [43].

The principles of user-centered design in developing VR/AR systems have been considered in 3(15%) studies [12, 34, 45].

The usability of rehabilitation systems was assessed in 8 studies (40%) using a usability questionnaire. The results of these studies showed an acceptable level of usability [12, 37, 38, 40, 41, 43, 47, 53]. Two studies used the system usability scale (SUS) questionnaire[38, 43], and one applied the single ease question (SEQ) test to record the user comments [40].

### Quality evaluation of studies

The quality evaluation results of studies based on the StaRI statement are presented shown in Table 3, and the results are summarized as follows:

**Table 3:** Evaluation of the studies according to the StaRI statement

StaRI standard	Title and abstract		Introduction				Methods: description							Methods: evaluation				
	Title	Abstract	Introduction	Rationale		Aims	Design	Context	Targeted ‘sites’		Description		Sub-groups	Outcomes		Process evaluation	Economic evaluation	
				S	I				S	I	S	I		S	I		S	I
1. Guggenberger et al. [31]	✓	✓	✓	✓	✓	✓	✓	✓	✓	✓	✓	✓	✓	✓	✓	✓	✘	✘
2. Zhao et al. [32]	✘	✓	✓	✓	✓	✓	✓	✓	✘	✘	✓	✘	-	✓	✘	✓	✘	✘
3. Prvu Bettger et al. [33]	✘	✓	✓	✓	✓	✓	✓	✓	✓	✓	✓	✓	-	✓	✓	✓	✘	✓
4. Günaydin and Arslan [12]	✓	✘	✓	✘	✘	✓	✓	✓	✓	✓	✓	✘	✓	✓	✓	✓	✘	✘
5. Perez Medina et al. [34]	✓	✓	✓	✓	✘	✓	✓	✓	✘	✘	✓	✘	-	✓	✓	✓	✘	✘
6. Kontadakis et al. [35, 36]	✘	✓	✓	✓	✓	✓	✓	✓	✓	✓	✓	✓	✘	✓	✓	✓	✘	✘
7. Karayigit and Celikcan [37]	✘	✓	✓	✓	✘	✓	✓	✓	✘	✘	✓	✘	-	✓	✓	✓	✘	✘
8. Rybarczyk et al. [38]	✘	✓	✘	✓	✓	✓	✓	✓	✘	✘	✓	✘	-	✓	✘	✓	✘	✘
9. Feng et al. [39]	✘	✓	✓	✓	✘	✓	✓	✓	✘	✘	✓	✘	-	✓	✓	✓	✘	✘
10. Pruna et al. [40]	✘	✓	✓	✓	✘	✓	✓	✓	✘	✓	✓	✘	-	✓	✓	✓	✘	✘
11. Tannous et al. [41, 42]	✓	✓	✓	✘	✘	✓	✓	✓	✘	✘	✓	✘	-	✓	✓	✓	✘	✘
12. Su and Cheng [43]	✓	✘	✓	✓	✓	✓	✘	✓	✘	✓	✓	✓	-	✓	✓	✓	✘	✘
13. Zhiyu et al. [44]	✘	✘	✓	✓	✘	✓	✓	✓	✓	✘	✓	✘	-	✓	✘	✓	✘	✘
14. Antón et al. [45]	✘	✓	✓	✓	✓	✓	✓	✓	✓	✓	✓	✓	-	✓	✓	✓	✘	✘
15. Choi et al. [46]	✓	✓	✓	✓	✓	✓	✓	✓	✘	✘	✘	✘	-	✓	✓	✓	✘	✘
16. Gonzalez-Franco et al. [47]	✘	✓	✓	✘	✘	✘	✓	✓	✓	✓	✓	✓	-	✓	✓	✓	✘	✘
17. Garcia and Felix Navarro [48]	✘	✘	✓	✓	✘	✓	✓	✓	✘	✘	✓	✘	-	✘	✘	✓	✘	✘
18. Patanapanich et al. [49]	✘	✓	✓	✘	✘	✓	✓	✓	✘	✘	✓	✘	-	✓	✘	✓	✘	✘
19. ^a^Shih-Ching et al. [50]; Yeh et al. [51]	✘	✓	✓	✓	✓	✓	✓	✓	✓	✓	✓	✓	-	✓	✓	✓	✘	✘
20. Maurer et al. [52]	✘	✘	✓	✘	✘	✘	✘	✓	✘	✘	✘	✘	-	✘	✘	✘	✘	✘

StaRI standard	Methods: evaluation			Results										Discussion			General
	Sample size	Analysis	Sub-group analyses	Characteristics	Outcomes		Process outcomes	Economic evaluation		Sub-group analyses	Fidelity/ adaptation	Contextual changes	Harms	Structured discussion	Implications		Statements
					S	I		S	I						S	I	
1. Guggenberger et al. [31]	✓	✓	✘	✓	✓	✓	✓	✘	✘	-	✓	✘	✓	✓	✓	✓	✓
2. Zhao et al. [32]	✘	✓	-	✘	✓	✘	✓	✘	✘	-	✘	-	✓	✓	✘	✘	✘
3. Prvu Bettger et al. [33]	✓	✓	-	✓	✘	✓	✓	✘	✓	-	✓	-	✘	✓	✘	✘	✓
4. Günaydin and Arslan [12]	✓	✓	✓	✓	✘	✓	✘	✘	✘	✓	✘	-	✘	✘	✘	✘	✘
5. Perez Medina et al. [34]	✘	✓	-	✘	✘	✘	✓	✘	✘	-	✓	-	✘	✓	✓	✘	✘
6. Kontadakis et al. [35, 36]	✓	✓	✘	✘	✓	✓	✓	✘	✘	✘	✓	✓	✓	✘	✘	✘	✘
7. Karayigit and Celikcan [37]	✓	✓	-	✓	✓	✓	✘	✘	✘	-	✘	-	✘	✘	✘	✘	✘
8. Rybarczyk et al. [38]	✓	✓	-	✓	✓	✘	✓	✘	✘	-	✘	-	✘	✘	✘	✘	✘
9. Feng et al. [39]	✓	✓	-	✓	✓	✓	✘	✘	✘	-	✘	-	✘	✓	✘	✘	✘
10. Pruna et al. [40]	✓	✓	-	✓	✓	✓	✘	✘	✘	-	✘	-	✘	✘	✘	✘	✘
11. Tannous et al. [41, 42]	✓	✓	-	✓	✓	✓	✘	✘	✘	-	✘	-	✘	✓	✘	✘	✓
12. Su and Cheng [43]	✓	✓	-	✓	✘	✓	✓	✘	✘	-	✘	-	✘	✘	✘	✘	✓
13. Zhiyu et al. [44]	✘	✘	-	✓	✓	✘	✓	✘	✘	-	✓	-	✓	✘	✘	✘	✘
14. Antón et al. [45]	✓	✓	-	✓	✓	✓	✓	✘	✘	-	✓	-	✘	✓	✓	✓	✓
15. Choi et al. [46]	✓	✘	-	✘	✓	✓	✘	✘	✘	-	✘	-	✘	✓	✘	✘	✘
16. Gonzalez-Franco et al. [47]	✓	✓	-	✘	✘	✓	✘	✘	✘	-	✘	-	✘	✘	✘	✘	✘
17. Garcia and Felix Navarro [48]	✘	✘	-	✘	✘	✘	✘	✘	✘	-	✘	-	✘	✘	✓	✘	✘
18. Patanapanich et al. [49]	✓	✓	-	✓	✓	✓	✓	✘	✘	-	✓	-	✘	✘	✘	✘	✘
19. ^a^Shih-Ching et al. [50]; Yeh et al. [51]	✓	✓	-	✓	✘	✓	✘	✘	✘	-	✘	-	✘	✘	✘	✘	✘
20. Maurer et al. [52]	✘	✘	-	✘	✘	✘	✘	✘	✘	-	✘	-	✘	✘	✘	✘	✘

^a^A key concept of the StaRI standards is the dual strands of describing the implementation strategy and, on the other, the clinical intervention that is being implemented. These strands are represented as two columns in the checklist, indicated by S and I

In the title and summary section, 85% of the studies had followed at least one of the two checklist items. In the introduction section, 75% of the study had completed at least two of the three items mentioned in the checklist. In the method section (description), 50% of the studies had at least 4 out of 7 checklist items. In the method (evaluation) part, 80% of the survey had observed at least half of the items mentioned in this part of the checklist. But in the economic sector, no study had fully observed this case. In the results section, only 30% of the studies presented at least 5 of the ten items mentioned in the checklist. And in the discussion section, 15% of the studies presented at least two of the three items mentioned in the checklist.

Based on the results of the qualitative analysis of studies according to the StaRI statement, two studies evaluated the VR/AR rehabilitation system through RCT. Yeh et al.’s study included an experimental group with a computer game and a control group with traditional rehabilitation. For the experimental group, during the experiment, individuals can check the rehabilitation status in real-time through the system and learn the next movement mode. For the control group, rehabilitation dominated the rehabilitation process, including bending the knee and raising the thigh [51]. Prvu Bettger et al. [33] conducted an RCT to evaluate the effect of a virtual PT program on total costs at 12 weeks post-TKA and to evaluate the clinical efficacy and safety of virtual PT over conventional care with traditional PT. Patients in the intervention group used the VERA system, and patients in the routine care group followed the recommendations of their care team for all medical and pre and post-operative rehabilitation care.

According to Table 3, the StaRI checklist items for the studies are interpreted as follows:

- Title and abstract

The titles of 14 (70%) studies were poorly reported [32, 33, 35, 37–40, 44, 45, 47–49, 52, 57], and the study methodology was not mentioned in the title. However, the abstract of the studies was of acceptable quality.

- Introduction

The introduction to the studies was of acceptable quality, except for the investigation by Rybarczyk et al.[38]. They did not clearly state some parts of the introduction, including the description of the problem.

- Methods

In the method section, most studies were well-designed in design and content. Still, as shown in Table 3, the studies were weak regarding targeted ‘sites’ and the description of the intervention and the implementation strategy. In addition, the evaluation section of the studies was presented acceptably, but none of the studies performed an economic evaluation.

- Result

In the results section, the outcome of 11 (55%) studies, i.e., fidelity to implementation strategy as planned, and essential harms or unintended effects in each group were not well expressed [37–41, 43, 46–48, 52, 57].

- Discussion

In the discussion section, 12 (60%) studies did not express the findings, strengths, limitations, and comparisons with other studies [12, 35, 37, 38, 40, 43, 44, 47–49, 52, 57]. In addition, the research concepts were not discussed in 16 (80%) studies [12, 32, 33, 35, 37–41, 43, 44, 46, 47, 49, 52, 57].

## Discussion

This systematic review was performed to identify the software and hardware infrastructure of VR/AR-based systems and identify the factors necessary for efficient reporting for these systems. A total of 20 studies on VR/AR-based systems for people with lower limb disorders were included. Nearly half of the studies used the Kinect sensor as a VR input device and the Unity game engine for visualization. Some literature has enumerated various techniques for analyzing the movements and evaluating the system’s usability. Also, in terms of reporting quality, the results based on the StaRI checklist showed poor reporting in the study’s title and discussion.

Lower limb rehabilitation using commercially available VR/AR-based rehabilitation systems is unsuitable for everyone as they are not tailored for people with specific medical disorders. The systems developed based on determining the functional needs of patients and mobility requirements are highly adapted to the needs of different groups of patients [27]. In the studies reviewed, all VR/AR-based rehabilitation systems were custom-made. This is a strength of the studies because it allows the customization of movements and exercises based on patient characteristics.

In the following section, the research findings, which are based on the purposes of the study, are mentioned:

### Technology

Many types of sensors and technologies for tracking human movement are used in VR/AR-base systems; the most important ones include 1. Optical systems; 2. Electrogoniometers; 3. Magnetic systems; and 4. Inertial system [35, 36]. According to the results of the present study, the Kinect sensor, which is an optical system, has been used in studies primarily for motion tracking which can have several reasons:

- First, many studies have shown this sensor’s high potential and accurate motion detection [57, 58]. A comparison of motion detection between Kinect and optical motion capture shows that Kinect can accurately detect the connection angle with a minor error of less than 2 [49]. Also, the study by Dajime et al. [57] showed that Kinect-based automatic motion assessment was a convenient, new, and practical approach to assessing movement. In another study, Mousavi Hondori et al. [59] reviewed the impact of Kinect on rehabilitation; they discovered that Kinect was an acceptable tool for rehabilitation due to its low cost, reasonable accuracy, and acceptance by patients and therapists. Because of the computational load required to extract the human skeleton from an RGB image, building a real-time interactive system using contrast-based imaging is unreliable. In contrast, developers prefer deep imaging devices such as the Kinect because they offer an SDK that accesses skeletal tracking data.
- The second reason is that the Kinect sensor’s performance can be increased by using machine learning models [57]. Although the validity of the Kinect position data has been much debated, its reliability and compatibility are undeniable.
- A third reason to be interested in using Kinect could be that users who do not need assistive devices when using it; in addition, this cost-effective and portable sensor allows detection of the anatomy of a patient’s body parts [35].

In general, rehabilitation system developers should choose their depth sensors according to their needs and problems, but the Kinect is fine when full-body movement is required [59]. However, this sensor has limitations that can affect its ability to detect motion, such as shadow, room light, subject size, clothing, gesture, and distance from the Kinect. Algorithms are needed to filter the data received from the sensor to eliminate random noise before using the data for processing [60]. In addition, when it is essential to accurately identify the angle of the legs, the combination of Kinect and sensors such as IMU can be used.

### Movement recognition and assessment

Human movement recognition is challenging because of the complex posture made by humans [61]. There are various approaches to sensor technologies and computational algorithms in this field. Movement recognition is done through a process flow, which involves recording and filtering the raw data, extracting the features, and classifying using machine learning models [55].

Generally, movement recognition approaches can be roughly divided into two categories: (1) pattern-based and (2) rule-based.

The pattern-based approach compares the measured motion with the reference motion pattern. The reference pattern is usually obtained by performing the correct exercises by healthy people. There are several methods for calculating the similarity between the movement performed by patients and the reference pattern, for example, obtaining connection angles at a set of characteristic points, DTW.

- Automatic construction of the model using sample data and the possibility of evaluating new types of exercises can be the reason for choosing this method in studies.

The rule-based approach uses a set of rules for an exercise defined by an expert. These rules are used as a standard for assessing the accuracy of movement.

- The rule-based approach does not require the registration of samples and the construction of dynamic models. Still, the disadvantage of this method is that the rules are related to one exercise and cannot be used for other activities [62].

### VR/AR systems development tools

As stated in the results, most studies have chosen the Unity 3D game engine as a powerful and highly flexible tool for developing a platform for serious rehabilitation games.

Unity is popular because of its support, fast prototyping capability, and compatibility with most commercial VR /AR displays and interaction tools [63]. Furthermore, game development and execution can be done on operating systems such as Linux, Windows, and Mac OS [42].

### Evaluation of VR/AR-base systems

To maximize the benefits of VR/AR technologies, developers, policymakers, and the organizations that implement them must consider the types of user needs from the beginning. During the development of a VR/AR system, it is essential to focus on user-based design because, in the real world, the developer will evaluate the system with the real user, using it when the user is satisfied with your program or system [64]. Despite the importance of user-centric design principles in developing VR / AR systems, this method was included in only 3 (15%) studies [12, 34, 45].

Moreover, it is imperative to evaluate the degree of usability, especially when transferring such systems from a research laboratory to clinical use, because they are used in clinical care [65]. Standard methods for usable studies are well documented and described in the literature [66]. However, few guidelines exist on selecting and performing usability assessments for VR/AR health interventions compared to other digital health technologies. The results of this study show different and non-specific methods for evaluating the usability of VR systems.

Zhang et al. [65] provided the readers with a list of usability assessment resources to solve this problem. They described six categories of usability assessment for virtual environments, including 1) cognitive or task walkthrough, 2) graphical evaluation, 3) post hoc questionnaires or interviews, 4) physical performance evaluation, 5) user interface evaluation, and 6) heuristic evaluation. The recommendations in their study can be helpful for better performance of health-related VR systems.

In addition, we recommend ISO 9126–1 as a standard reference to measure the quality aspects of a system. This is because evaluation aspects such as performance, reliability, usability, efficiency, maintainability, and portability can be evaluated [64].

### Quality evaluation of studies

Implementation studies are often poorly reported and indexed. This reduces the potential of these studies to inform about the provision of health care services, reproducibility, and generalizability. To improve the VR/AR-based rehabilitation systems, a good description of the functions and features of the system is essential. Therefore, to better design and report interdisciplinary studies and assist researchers in this field, we conducted a qualitative review using the StaRI checklist. The StaRI aims to provide guidelines for a clear and accurate report of executive studies, which is why we reviewed the compliance of the selected studies with this standard and made recommendations based on the results obtained:

- Title and abstract

Considering methodological studies are not usually well elaborated in the title section, we recommend it is more appropriate to mention the methodology to increase the reporting quality in the study’s title. Likewise, in the abstract section, the study’s results should be stated clearly, in addition to stating the implementation strategy.

•Introduction

In the introduction section, in addition to describing the problem and the reason for choosing the strategy, it is recommended to focus on expressing the validity of the ongoing intervention and evidence for its effectiveness.

•Methods

Some studies have focused on just the implementation section or the clinical section. According to the results, the sections on the targeted site and the intervention strategy and implementation recommendations in the studies were not of acceptable quality. The characteristics of the targeted site (such as location/personnel/resources) for implementation, the intervention’s target population, and any eligibility criteria should be stated. In addition, studies should clearly outline the treatment protocol, some sessions, and treatment intervals, as well as the implementation strategy, stakeholder participation, and strategy processes in the implementation strategy section. Furthermore, the use of resources, costs, economic results, and analysis to implement the intervention were examined in only one study [33]. One of the main limitations of using VR/AR technology as a clinical tool involves associated costs. Reducing the costs in the health care systems could be a major argument against using VR/AR technology in clinical facilities [67]. Therefore, launching rehabilitation services based on VR/AR cost–benefit field research seems necessary.

- Result

The results of most studies were not well expressed, so we suggest that to improve the quality of the studies report, we need to consider the possible side effects of clinical interventions using VR/AR, professional training, and utilization of review templates.

- Discussion

Most studies systematically describe the process of implementing strategies but do not discuss the concept behind these steps and strategies. Researchers should focus on summarizing the findings to present practical reports in the future, expressing their experiences, and comparing them with the results of previous studies and the challenges in the discussion section.

### Strengths and limitations

The current review had three main features. First, a systematic review was performed using defined research questions according to the PRISMA guidelines. Second, the selected studies were evaluated based on the StaRI checklist for each survey. Third, the methods and algorithms used to assess the accuracy of patient performance, the tools used to develop custom rehabilitation systems, and the usability of VR/AR technology can be used in lower limb rehabilitation, which can be a roadmap for the development and implementation of such systems in the future. However, there were a few limitations. First, only articles in English were included. Articles published in languages other than English may contain valuable information not covered in this article. Second, most of the studies reviewed included only asymptomatic volunteers; therefore, it is impossible to give a definite opinion about the effectiveness of the developed systems for lower limb disorders. Third, one of the strengths of this research was an evaluation of the quality of published articles. However, since it was impossible to quantify the quality of the article in the checklist, comparing them based on quality was impossible. Finally, all studies included implementing an interactive VR/AR rehabilitation system for the lower limbs, and studies with a complete focus on balance were excluded.

## Conclusion

Our findings show that academic studies do not provide a detailed description of the technical aspects of developed VR/AR-based rehabilitation systems. They only describe the type of sensor and system implementation tool. Therefore, it is not possible to reuse the developed rehabilitation systems. It is essential to provide a good description and report of how these systems are developed to reduce this problem. We believe that reporting statements like StaRI can provide better-quality studies to guide future researchers in this field.

## Data Availability

All data generated or analyzed during this study are included in this published article.

## Abbreviations

VR: Virtual Reality
AR: Augmented Reality
ICT: Information and Communication Technologies
IEEE: Institute of Electrical and Electronics Engineers
MeSH: Medical Subject Headings
PRISMA: Preferred Reporting Items for Systematic Reviews and Meta-Analyses
ROM: Range of Motion
DTW: Dynamic Time Warping
StaRI: Standards for Reporting Implementation
WHO: World Health Organization
IMU: Inertial Measurement Units
EMG: Electromyogram
3D: Three-Dimensional
HMD: Head-Mounted Displays
SEQ: Single Ease Question
SUS: System Usability Scale
TKR: Total Knee Replacement
THR: Total Hip Replacement
ACL: Anterior Cruciate Ligament
OLST: One-Leg Standing Test
KOOS: Knee injury and Osteoarthritis Outcome Score
PROMIS: Patient-Reported Outcomes Measurement Information System
AKSS: American Knee Society Score
SDK: Software Development Kit
OA: Osteoarthritis
KNN: K-Nearest Neighbour
PCA: Principal Component Analysis
SVM: Support Vector Machines
XML: Extensible Markup Language
RGB: Red, Green, and Blue
ANFIS: Adaptive Network-based Fuzzy Inference System
ISO: International Organization for Standardization
PT: Physical Therapy
SRPS: Self-Physical Rehabilitation System
RehApp: Rehabilitation Application
KiReS: Kinect Rehabilitation System
HE: Hough Leg Angle Estimator
PU: Perceived Usefulness
PE: Perceived Ease of use
AT: Attitude
IU: Intention of use
MVC: Model-View-Controller
AI: Artificial intelligence
SKRM: Standing Knee Raises Movement
SSP: Syringe Service Program
RMS: Mean square root
